# Serum neurofilament light is superior to glial fibrillary acidic protein to distinguish sporadic frontotemporal dementia from late-onset primary psychiatric disorders: a retrospective DIPPA-FTD study

**DOI:** 10.1136/bmjno-2024-001007

**Published:** 2025-06-01

**Authors:** Sterre Catharina Maria de Boer, Chiara Fenoglio, Andrea Arighi, Lisa Wissink, Lina Riedl, Ishana Rue, Ramon Landin-Romero, Sophie Matis, Zac Chatterton, Glenda M Halliday, Janine Diehl-Schmid, Olivier Piguet, Inge M W Verberk, Charlotte E Teunissen, Simon Ducharme, Sven J van der Lee, Yolande A L Pijnenburg, Daniela Galimberti

**Affiliations:** 1 Alzheimer Center Amsterdam, Neurology, Vrije Universiteit Amsterdam, Amsterdam UMC location VUmc, Amsterdam, Noord-Holland, The Netherlands; 2 Department of Psychology, The University of Sydney Brain and Mind Centre, Camperdown, New South Wales, Australia; 3 Amsterdam Neuroscience, Neurodegeneration, Amsterdam, Noord-Holland, The Netherlands; 4 University of Milan, Milano, Italy; 5 Fondazione IRCCS Ca’ Granda Ospedale Maggiore Policlinico, Milan, Lombardia, Italy; 6 Psychiatry and Psychotherapy, Klinikum rechts der Isar, Technische Universität München, Munich, Germany; 7 Douglas Mental Health University Institute, Department of Psychiatry, McGill University Montreal, Montreal, Quebec, Canada; 8 School of Psychology, Brain & Mind Centre, The University of Sydney, Sydney, New South Wales, Australia; 9 Brain and Mind Centre, Faculty of Medicine and Health, University of Sydney, Sydney, New South Wales, Australia; 10 Psychiatry, Technical University of Munich School of Medicine, Munchen, Bayern, Germany; 11 Clinical Center for Psychiatry, kbo-Inn-Salzach-Klinikum gemeinntzige GmbH, Wasserburg am Inn, Bayern, Germany; 12 Brain and Mind Centre, The University of Sydney, Sydney, New South Wales, Australia; 13 Neurochemistry Laboratory, Department of Clinical Chemistry, Amsterdam UMC—VUMC location, Amsterdam, The Netherlands; 14 Neurochemistry Laboratory, Clinical Chemistry Department, Amsterdam Neuroscience, Vrije Universiteit Amsterdam, Amsterdam, Noord-Holland, The Netherlands; 15 Psychiatry, Douglas Mental Health University Institute, Montreal, Quebec, Canada; 16 McConnell Brain Imaging Centre, Montreal Neurological Institute-Hospital, Montreal, Quebec, Canada; 17 IRCCS Ospedale Maggiore Policlinico, Milan, Italy

**Keywords:** NEUROPSYCHIATRY, FRONTOTEMPORAL DEMENTIA, PSYCHIATRY

## Abstract

**Background:**

Sporadic behavioural variant frontotemporal dementia (bvFTD) is often misdiagnosed as late-onset primary psychiatric disorder (PPD). Previous research in small sample sizes has shown that neurofilament light (NfL) and glial fibrillary acidic protein (GFAP) are promising biomarkers to distinguish FTD from PPD. We aimed to investigate the discriminative value of NfL and GFAP in a multicentre cohort of sporadic bvFTD and late-onset PPD.

**Methods:**

In total, n=275 sporadic bvFTD and n=82 PPD were included from our DIPPA-FTD study. Baseline serum NfL and GFAP levels were measured using Simoa. Biomarker levels were compared between groups. The effect of age and sex on NfL and GFAP was measured using linear regression models. Discriminative accuracies were assessed using logistic regression models and receiver operating characteristic curves, corrected for age and sex. Within a subset of bvFTD patients who were deceased, the prognostic value of biomarkers was assessed by correlating disease duration (age at death minus age at blood sampling) with biomarker levels.

**Results:**

Significantly higher serum median NfL and GFAP levels were found in sporadic bvFTD (NfL 33.3 pg/mL, IQR (19.6–49.6); GFAP 124.5 pg/mL, IQR (83.5–181.6)) compared with PPD (NfL 12.2 pg/mL, IQR (9.8–17.9); GFAP 68.9 pg/mL, IQR (50.6–95.0), both p<0.001). Discriminative performance was AUC=0.872 for NfL, AUC=0.787 for GFAP and AUC=0.878 for NfL+GFAP (DeLong’s p for NfL+GFAP versus NfL AUCs: p=0.286). A shorter disease duration was significantly correlated with higher NfL, but not GFAP.

**Conclusion:**

Our study found that serum GFAP does not provide additional value as a discriminative marker compared with serum NfL alone when differentiating sporadic bvFTD from late-onset PPD.

**WHAT IS ALREADY KNOWN ON THIS TOPIC**

- Previous small-scale studies suggest neurofilament light (NfL) and glial fibrillary acidic protein (GFAP) as promising biomarkers for distinguishing behavioural variant frontotemporal dementia (bvFTD) from primary psychiatric disorders (PPD) developed later in life. However, these studies often included small sample sizes and both genetic and sporadic bvFTD cases, despite reported differences in biomarker levels. Therefore, our aim is to assess the discriminative value of NfL and GFAP in a large multicentre cohort focusing on sporadic bvFTD and late-onset PPD.

**WHAT THIS STUDY ADDS**

- Serum NfL is diagnostically useful in discriminating between bvFTD and late-onset PPD, while GFAP is inferior and does not significantly improve diagnostic performance beyond using NfL alone.

**HOW THIS STUDY MIGHT AFFECT RESEARCH, PRACTICE OR POLICY**

- These results imply a preference for clinically implementing serum NfL level as a diagnostic marker to distinguish sporadic bvFTD from late-onset PPD when in diagnostic doubt.

## Background

 The behavioural variant of frontotemporal dementia (bvFTD) is the second most common cause of young-onset dementia and is characterised by progressive decline in social cognition, behaviour, language and executive function.^1^ Early in the disease course, bvFTD may present with a spectrum of neuropsychiatric symptoms, including apathy, compulsiveness and delusions that often resemble features of primary psychiatric disorders (PPD) like depression, obsessive–compulsive disorder and psychosis.^2^,^4^ The symptomatic similarities between bvFTD and PPD frequently pose a diagnostic challenge for clinicians, particularly since both conditions may manifest at a similar age, resulting in a diagnostic delay for bvFTD extending up to 6 years and a misdiagnosis rate ranging from 25% to 50%.^23 5^,^7^

Around 20%–30% of bvFTD cases have a mono-genetic, pathogenic cause.^8 9^ In the majority of bvFTD cases, often referred to as sporadic bvFTD, no mono-genetic causes are found, nor is there a familial history for FTD and related disorders. ^10^The absence of a diagnostic genetic marker in sporadic bvFTD amplifies the diagnostic challenge of distinguishing bvFTD from PPD, further complicating accurate identification and withholding adequate treatment and support, which causes major harm to patients and their families.^11^ With the current development of disease-modifying treatments, there is a growing imperative to diagnose sporadic bvFTD in its earliest disease stages, enabling trial participation opportunities in the future. Consequently, the pivotal challenge now lies in effectively distinguishing sporadic bvFTD from its primary differential diagnosis: late-onset PPD.^12^

Blood-derived biomarkers, such as neurofilament light (NfL) and glial fibrillary acidic protein (GFAP), could play a role in the diagnostic challenge currently faced in sporadic bvFTD. NfL is an abundant scaffolding protein, and concentrations increase in both cerebrospinal fluid and blood on neuro-axonal damage in neurodegenerative, traumatic, vascular and inflammatory diseases.^13^ GFAP is one of the cytoskeletal filament proteins in astrocytes, and its production and release are increased on reactive astrocytosis as a consequence of neurodegeneration.^14 15^ Although NfL and GFAP are cross-disease markers, rather than disease specific, there is emerging evidence that both NfL and GFAP can be of diagnostic value for differentiating bvFTD from other dementia types^15 16^ and from PPD.^17^,^23^ Despite the significance of these investigations, they often featured small sample sizes and included both genetic and sporadic bvFTD. Recent evidence suggests that NfL levels may differ between genetic and sporadic bvFTD,^24^ highlighting the need for focused investigation into genetically sporadic cases. By conducting a retrospective study with a large multicentre neuropsychiatric cohort, we here seek to evaluate the discriminative and combined value of serum NfL and GFAP levels, specifically in distinguishing sporadic bvFTD from late-onset PPD. In addition to evaluating its diagnostic value, we investigated the effect of age and sex on biomarker levels. In a subset of deceased sporadic bvFTD patients, we assessed the prognostic value of NfL and GFAP.

## Methods

### Participants

Subjects included participants of the retrospective DIPPA-FTD study. The retrospective DIPPA-FTD study is a multicentre cohort study. Specific inclusion and exclusion criteria, clinical assessments and diagnostic procedures have been described previously.^25^ In short, the study included participants from the Alzheimer Centre Amsterdam, the Brain and Mind Centre at the University of Sydney, the Douglas Mental Health University Institute at McGill University, Fondazione Ca’ Granda, IRCCS Ospedale Maggiore Policlinico and the Technical University of Munich. The retrospective DIPPA-FTD study included individuals aged 45 years and above, presenting with late-onset behavioural change, eventually diagnosed with bvFTD or late-onset PPD, meeting one of the Diagnostic and Statistical Manual of Mental Disorders classifications for major depressive disorder, manic episode, bipolar disorder, schizophrenia, personality disorders, delusional disorder or obsessive–compulsive disorder.^26^ BvFTD patients adhered to diagnostic criteria for probable or definite bvFTD (based on pathology),^1^ with a Clinical Dementia Rating score of ≤1 at baseline visit.^27^ Sporadic bvFTD was specified as having a Goldman score of ≥3^28 29^ or a Wood score of ‘Low’ or ‘Apparent sporadic’^30^ in the absence of a *C9ORF72* repeat expansion. Participants with a Goldman score of 2 or a Wood score of ‘Medium’ were included if genetic testing for *C9ORF72*, *GRN* and *MAPT* was negative. Individuals with a Goldman score of 1 and Wood score ‘High’ were excluded. In the case of individuals diagnosed with PPD, screening for the presence of *C9ORF72* repeat expansion had to yield negative results. Demographical and clinical data from the retrospective DIPPA-FTD dataset were retrieved, including age, sex, age at death, clinical subtype, and, if available, Alzheimer’s disease (AD) biomarker status positive/negative (eg, amyloid PET-scan or determination via amyloid-beta and p-tau in cerebrospinal fluid).

For this study, patients exhibiting amyotrophic lateral sclerosis cosymptomatology at the first visit were excluded from the current analyses (n=13), since any individual with a differential diagnosis of bvFTD versus PPD with manifestation of concomitant motor neuron disease eradicates diagnostic doubt. A total of 292 sporadic bvFTD patients and 86 late-onset PPD patients from the retrospective DIPPA-FTD dataset, with serum samples available, were included. Values exceeding three times the standard deviation (SD) from the mean were classified as outliers and removed from further analysis. This process was performed twice and separately for both NfL and GFAP values within the bvFTD and PPD group. Subsequently, these outliers (n=17 bvFTD and n=4 PPD) were excluded from further analysis.

### Blood-based biomarkers

At each site, non-fasted serum was collected through venepuncture at the first visit. The samples were centrifuged after a maximum of 2 hours, for ±10 min at 1800 x g at 4°C–20°C. The serum was subsequently aliquoted into 0.5 mL-portions in polypropylene storage tubes and stored at −80°C in the local biobanks until dry-ice transportation to the Neurochemistry Laboratory Amsterdam for measurement. Prior to analysis, samples were shortly thawed at room temperature and centrifuged at 10 000 g for 10 min. NfL and GFAP levels were measured in singlicates on the Simoa HDx analyser with the Simoa Neurology 4-plex E Kit (Product number: 103670; lot number: 503745; Quanterix, USA) according to kit instructions and with four times automated sample dilution. Calibration curves and quality control (QC) samples were measured in duplicates. Average intra-assay coefficient of variation of the two QCs measured in three runs was 11% for NfL and 10% for GFAP, and average interassay coefficient variation of these QCs over the three runs was 13% for NfL and 9% for GFAP. Serum NfL and GFAP levels had already been determined in 30 samples from the Amsterdam Dementia Cohort using the same procedure. Passing-Bablok analysis on n=6 (n=3 FTD, n=3 PPD) samples was used to harmonise the values obtained in those samples to the current batch (online supplemental material 1, figure S1A+S1B).

### Statistical analyses

Statistical analyses were conducted in R, V.4.2.1. Dichotomous data were compared between groups using χ^2^ tests, and continuous data were compared using independent t-tests or non-parametric tests (depending on normality). To investigate whether there is an effect of sex and age on biomarker level, we correlated log transformed NfL (logNfL) and GFAP (logGFAP) with age at blood sampling using Pearson correlation tests and fitted linear regression models including age and sex as independent and logNfL and logGFAP as dependent variables. We assessed the discriminative value of NfL and/or GFAP between bvFTD and PPD by fitting four separate logistic regression models (1) NfL, (2) GFAP, (3) NfL+GFAP and (4) GFAP/NfL ratio. All models were adjusted for sex and age at blood sampling. Receiver operating characteristic curves (ROC curves) were calculated together with area under the curve (AUC) values and compared between the four models using DeLong tests. To determine the optimal cut-off values for NfL and GFAP, two approaches were employed. Initially, absolute cut-off levels for NfL and GFAP were determined using the Youden index method without adjusting for age and sex. However, recognising the influence of age and sex on biomarker levels, we also used the adjusted model to determine probability cut-offs that optimise sensitivity and specificity while accounting for these covariates. Finally, in a subgroup of bvFTD patients who deceased, we investigated the prognostic value of NfL and GFAP. We calculated disease duration by subtracting the age at blood sampling from the age at death, which was correlated with the level of logNfL and logGFAP using Spearman correlation tests.

The level of statistical significance was set to p≤0.05.

## Results

**Figure 1:**
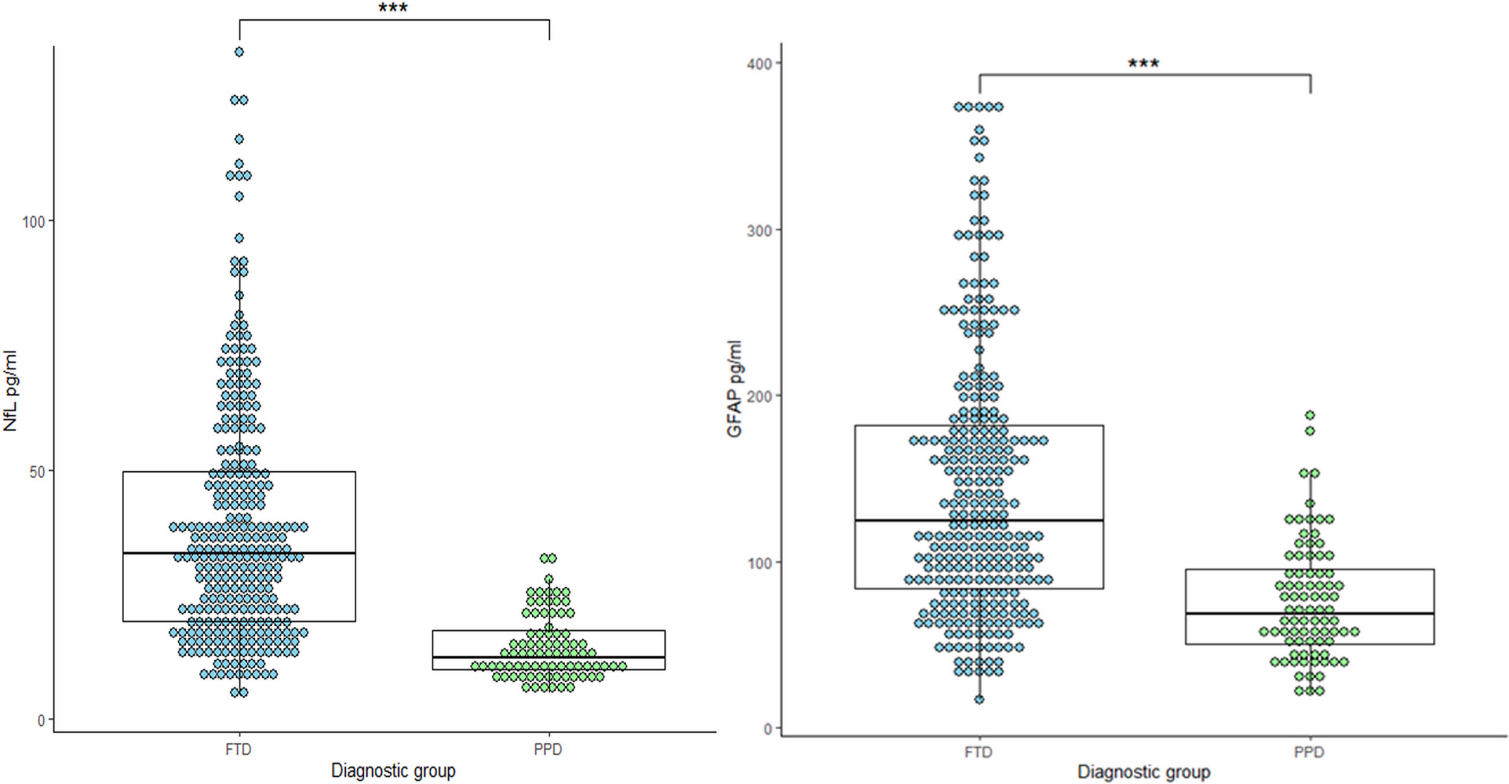
Boxplots of serum NfL and GFAP levels. Left: NfL pg/ml levels per diagnostic group: median serum NfL levels were significantly higher in the bvFTD group (NfL 33.3 pg/mL) compared with the PPD group (NfL 12.2 pg/mL, Mann-Whitney U, p<0.001). Right: GFAP pg/mL levels per diagnostic group: median serum GFAP levels were significantly higher in the bvFTD group (GFAP 124.5 pg/mL) than the PPD group (GFAP 68.9, Mann-Whitney U, p<0.001). FTD, sporadic behavioural variant of frontotemporal dementia; GFAP, glial fibrillary acidic protein; PPD, primary psychiatric disorder; NfL, neurofilament light. ***p<0.001.

The demographics are shown in table 1. There was a male predominance in both the bvFTD and PPD group, which did not differ between groups (p=0.412). The PPD group was significantly younger at blood sampling than the bvFTD group (mean age 59.6 years vs 65.1 years, p<0.001). Median serum NfL and GFAP levels were significantly higher in the bvFTD group compared with the PPD group (both p-values<0.001, figure 1). Mean and mean log-transformed biomarker levels, in addition to a visualisation of NfL and GFAP levels per diagnostic subgroups, can be found in the supplementary material (online supplemental material 2).

**Table 1:** Demographic table

	Sporadic bvFTD	PPD	P value
N, % total	275 (77.0)	82 (23.0)	NA
Subtype	Probable bvFTD n=264Definite bvFTD—TAU n=7Definite bvFTD—TDP n=4	Depression disorder n=57Bipolar disorder n=12Psychiatry NOS n=5Delusional disorder n=4Schizophrenia n=3OCD n=1	
Male, n (%)	172 (62.6)	56 (68.3)	0.412*
Age at sampling, mean (SD)	65.1 (8.70)	59.6 (7.0)	<0.001†
Symptom duration prior blood sampling, median years (IQR)	3.0 (2.0–5.0)	3.0 (2.0–5.0)	0.876
AD biomarkers, n ±	11/100	1/33	NA
NfL pg/mL, median (IQR)	33.3 (19.6–49.6)	12.2 (9.8–17.9)	<0.001‡
GFAP pg/mL, median (IQR)	124.5 (83.5–181.6)	68.9 (50.6–95.0)	<0.001‡
GFAP/NfL ratio, median (IQR)	4.0 (2.6–6.0)	5.4 (3.7–7.3)	<0.001‡

* Pearson χ2.

† Independent student t-test.

‡ Mann Whitney U test.

AD biomarkers, Alzheimer’s disease biomarkers positive (+) or negative (−) determined via cerebrospinal fluid or amyloid PET scan; bvFTD, behavioural variant of frontotemporal dementia; GFAP, glial fibrillary acidic protein; NfL, neurofilament light; NOS, not otherwise specified; OCD, obsessive compulsive disorder; PPD, primary psychiatric disorder.

### Effect of age and sex on biomarker levels

LogNfL and logGFAP levels correlated positively with the age at sampling in the total cohort (Pearson’s r=0.333, p<0.001 and r=0.488, p<0001, respectively, see online supplemental material 3 and 4). Within the bvFTD group, both logNfL (Pearson’s r=0.191, p=0.002) and logGFAP (Pearson’s r=0.441, p<0.001) were significantly correlated with age at sampling (online supplemental material S5A, S6A). Similarly, in the PPD group, logNfL (Pearson’s r=0.507, p<0.001) and logGFAP (r=0.378, p<0.001) were significantly correlated with age at sampling (online supplemental material 5 and 6).

Females with bvFTD had significantly higher NfL and GFAP levels than males with bvFTD (Mann-Whitney U, p-value=0.015, p-value<0.001, respectively, online supplemental material figure S7A,B). There were no significant differences in median NfL or GFAP between females with PPD and males with PPD (Mann-Whitney, p-value=0.156, p-value=0.800, respectively, online supplemental material 7). Linear regression analyses including predictor interactions (biomarker level as the dependent variable and diagnostic group * sex and diagnostic group * age as independent variables) showed a significant effect of diagnostic group, age at sampling, sex and the diagnostic group×sex interaction on log-transformed NfL levels (p-values: 0.002,<0.001, 0.002 and 0.041, respectively). However, the diagnostic group×age interaction was not significant (p=0.117). For log-transformed GFAP levels, significant effects were found for age at sampling, sex and the diagnostic group×sex interaction (p-values: <0.001, <0.001 and 0.004, respectively). In contrast, the diagnostic group and the diagnostic group×age interaction did not show significant effects (p-values: 0.519 and 0.618, respectively).

Linear regression analyses (biomarker level as dependent variable and sex and age as independent variables), stratified for diagnostic group showed that within the bvFTD group, a higher age at blood sampling (β=0.015, 95% CI (0.006 to 0.023), p<0.001) and being female (β=0.223, 95% CI (0.071 to 0.375), p<0.01) were significantly associated with higher logNfL levels. In the PPD group, a higher age at blood sampling was significantly associated (β=0.030, 95% CI (0.019 to 0.042), p<0.001) with a higher logNfL value, but there was no effect of sex (β=−0.092, 95% CI (−0.265 to 0.082), p=0.296). For GFAP, within the bvFTD group, higher age at blood sampling (β=0.030, 95% CI (0.023 to 0.037), p<0.001) and being a female (β=0.305, 95% CI (0.183 to 0.427) were significantly associated with higher logGFAP levels. In the PPD group, a higher age at blood sampling (β=0.026, 95% CI (0.012 to 0.040), p<0.001) was significantly associated with higher logGFAP levels, but there was no effect of sex (β=0.033, 95% CI (−0.177 to 0.243), p=0.753).

### Discriminative value NfL and/or GFAP between bvFTD and PPD

**Figure 2:**
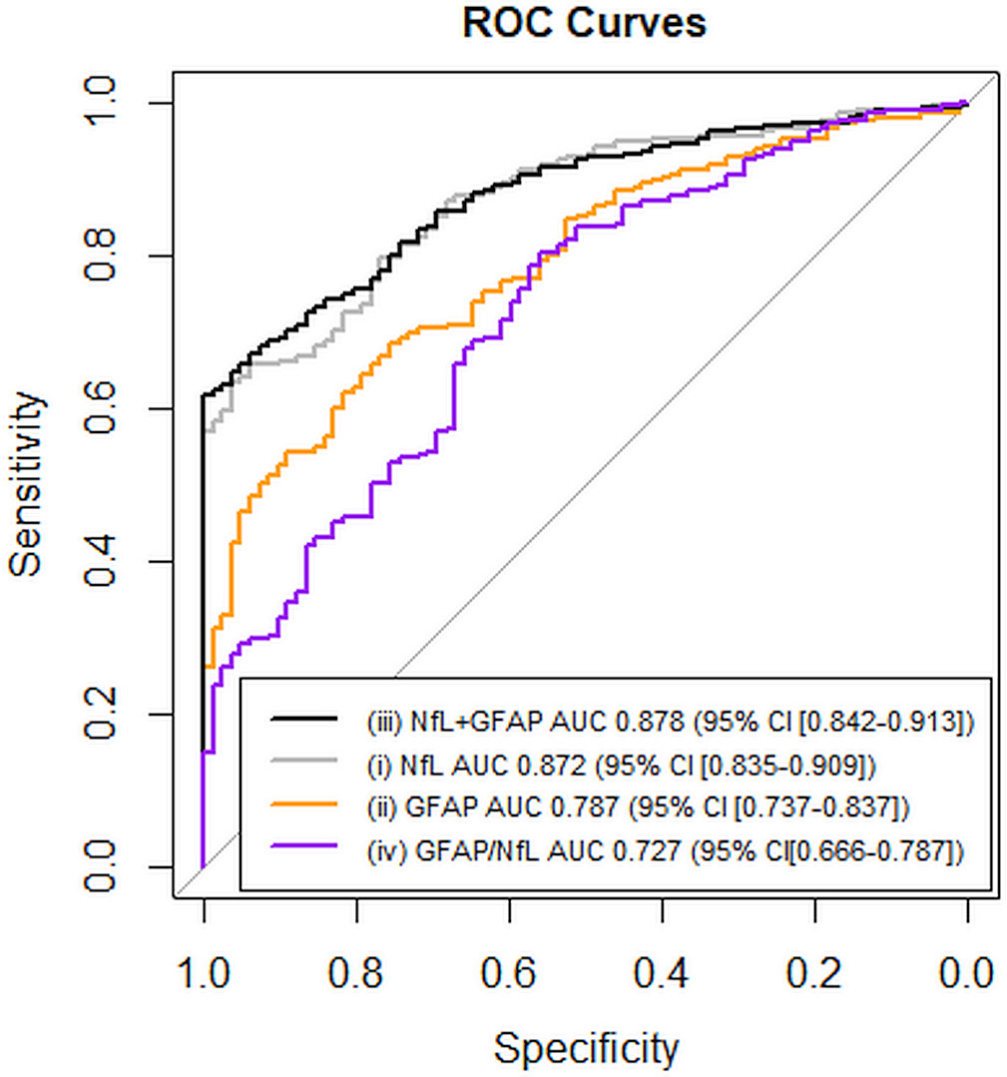
ROC curve analyses with AUC of each model. Grey line: model (1) NfL. Orange line: model (2) GFAP. Black line: model (3) NfL+GFAP. Purple line: model (4) GFAP/NfL. All models were adjusted for sex and age. The AUC value of (1) NfL 0.872 was significantly higher than (2) GFAP 0.787 (p<0.001) and (4) GFAP/NfL 0.727 (p<0.001). The AUC of (1) NfL was lower but not significantly different from (3) NfL+GFAP AUC 0.878 (p=0.286). AUC value of (2) GFAP 0.787 was significantly lower than (3) NfL+GFAP 0.878 (p<0.001) but not different from (4) GFAP/NfL 0.727 (p=0.095). The AUC of (3) NfL+GFAP 0.878 significantly differed from (4) GFAP/NfL 0.727 (p<0.001). AUC, area under the curve; GFAP, glial fibrillary acidic protein; NfL, neurofilament light; ROC, receiver operating characteristic curve.

Diagnostic performance was assessed for models (1) NfL, (2) GFAP, (3) NfL+GFAP and (4) GFAP/NfL ratio using logistic regression models, including age at sampling and sex. For model (1) NfL, one unit increase in NfL pg/mL was associated with 1.15 higher odds, which is equivalent to 27.34 higher odds with one SD increase in standardised NfL, of being in the bvFTD group than in the PPD group (p-value<0.001). In the age-adjusted and sex-adjusted ROC curve analysis between the bvFTD and PPD group, the AUC was 0.872 (95% CI (0.835 to 0.909)). Model (2) GFAP showed that one unit increase in GFAP pg/mL was associated with 1.02 higher odds, which is equivalent to a 4.90 higher odds with one SD increase in standardised GFAP, of being in the bvFTD group (p-value<0.001). The age-adjusted and sex-adjusted ROC curve analysis between bvFTD and PPD had an AUC of 0.787 (95% CI (0.737 to 0.837)). Combining NfL and GFAP in model (3) showed that both NfL (p-value<0.001) and GFAP (p-value=0.028) were significantly associated with higher odds of being in the bvFTD group, with the age-adjusted and sex-adjusted ROC curve analysis showing an AUC of 0.878 (95% CI (0.842 to 0.913)). Model (4) GFAP/NfL showed that one unit decrease in the ratio was significantly associated with higher odds of being in the bvFTD group (OR 1.18, p-value<0.001) and gave an AUC of 0.727 (95% CI (0.666 to 0.787), see online supplemental material 8 output logistic regressions). Hierarchical logistic regression analysis showed that adding GFAP to the adjusted model (1) significantly improved predictive performance (anova model (1) and model (3): p=0.02). Between standalone markers, (1) NfL alone had the highest AUC of 0.872, which did not significantly improve when GFAP was added to the model ((3): p=0.286, figure 2)).

The absolute cut-off value of NfL and GFAP without adjusting for age and sex was 25.9 pg/mL for NfL (sensitivity 65%, specificity 96%, positive predictive value 98%, negative predictive value 45%, positive likelihood ratio 16.3 and negative likelihood ratio 0.37) and 95.2 pg/mL for GFAP (sensitivity 68%, specificity 76%, positive predictive value 90%, negative predictive value 42%, positive likelihood ratio 2.8 and negative likelihood ratio 0.4). The Youden index probability cut-off value of NfL and GFAP, adjusted for age and sex, within our cohort was 0.873 for NfL, which led to the same sensitivity of 65% and specificity of 96%. The probability cut-off for GFAP was 0.750, which led to the same sensitivity of 68% and specificity of 76%.

### Prognostic value of NfL and GFAP in sporadic bvFTD

**Figure 3:**
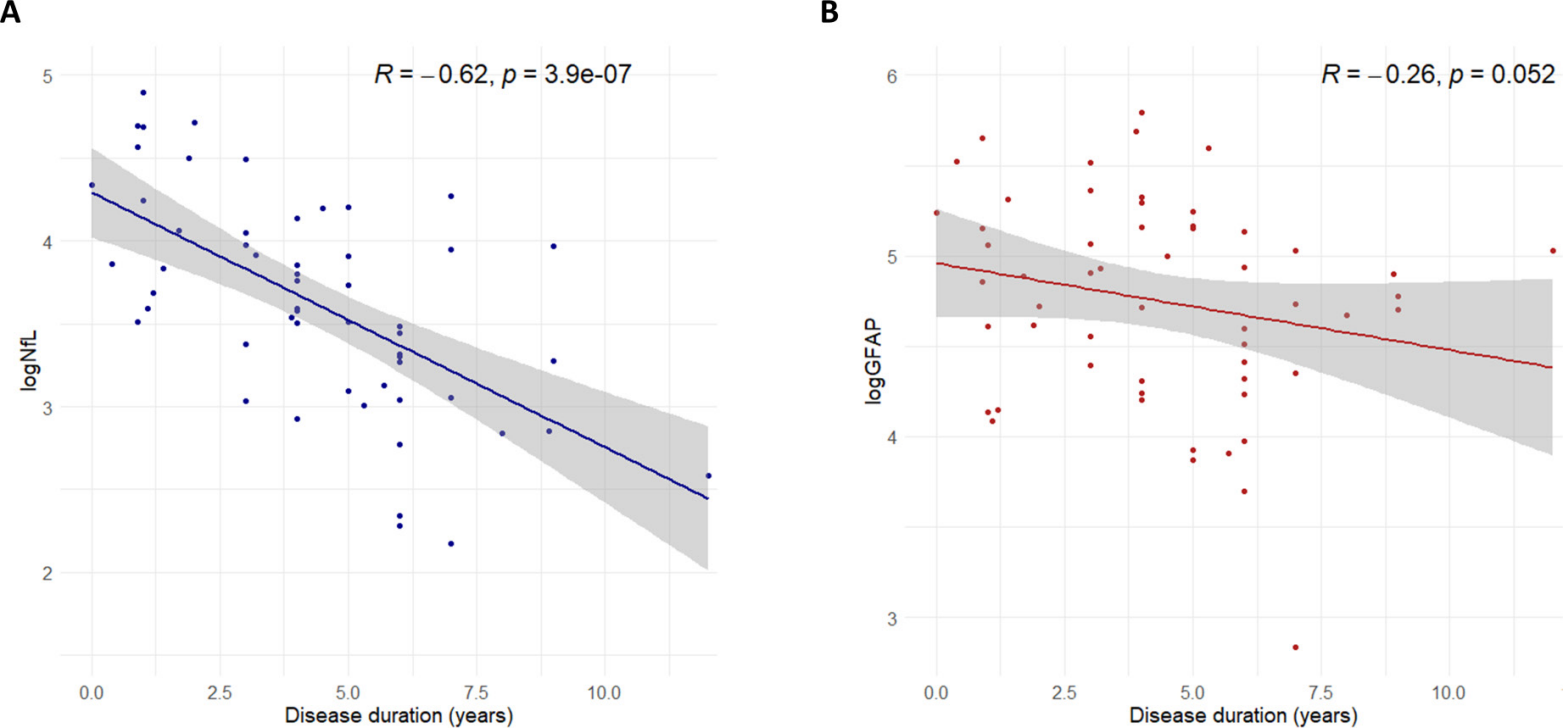
Scatterplot of (**A**) NfL and disease duration and (**B**) GFAP and disease duration. (**A**) Serum NfL level was significantly correlated with disease duration (Spearman’s rho=−0.618, p<0.001). (**B**) Scatterplot of GFAP and disease duration, serum GFAP levels were not significantly correlated with disease duration (Spearman’s rho=−0.216, p=0.052). Included in correlation analyses: bvFTD n=56. GFAP, Glial fibrillary acidic protein; NfL, Neurofilament light.

The prognostic value of NfL and GFAP in sporadic bvFTD was assessed by correlating the disease duration after blood sampling (ie, time between baseline visit and time of death) with NfL and GFAP values. Within the sporadic bvFTD group, n=56 cases were deceased and had information on age at death available. The median disease duration after blood sampling was 4.0 median (IQR 2.75–6.00) years. Serum NfL level was significantly inversely correlated with disease duration (Spearman’s rho=−0.618, p<0.001, figure 3A). No significant correlations between serum GFAP and disease duration were found (Spearman’s rho=−0.216, p=0.052, figure 3B).

## Discussion

In this study, we evaluated the diagnostic utility of serum NfL and GFAP levels to distinguish sporadic bvFTD from late-onset psychiatric disorders in a large multicentre neuropsychiatric cohort. Significantly higher levels of both serum NfL and GFAP were found in the sporadic bvFTD group compared with the late-onset PPD group. Although the addition of GFAP to NfL alone did significantly improve predictive performance, adding GFAP did not result in a statistically significant improvement of AUC value achieved by NfL alone. This suggests that NfL alone may offer the most optimal and cost-effective, discriminative performance when differentiating sporadic bvFTD from late-onset PPD. In addition to an effect of age, we observed an effect of sex on serum NfL and GFAP levels in the sporadic bvFTD group but not in the PPD group. Additionally, we found that within the sporadic bvFTD, baseline NfL but not GFAP levels correlated with disease duration in the period after blood sampling. These results altogether confirm the usefulness of NfL in the differential diagnosis between sporadic bvFTD and late-onset PPD and suggest that serum GFAP does not offer additional diagnostic or prognostic value compared with serum NfL alone. However, the potential complementary role of GFAP alongside NfL warrants further consideration.

To the best of our knowledge, this is the largest study to date on the diagnostic utility of NfL and GFAP in differentiating sporadic bvFTD from late-onset PPD. Studies independent from the cohorts included in our multicentre study, although containing both genetic and sporadic bvFTD cases, showed in agreement with our findings that blood-based NfL can discriminate between bvFTD and PPD with sensitivities ranging from 70% to 88% and specificities ranging from 85% to 93%, respectively.^18 20 21^ In our study, we identified an optimal cut-off for NfL at 25.9 pg/mL, demonstrating a sensitivity of 65% and specificity of 96%. Arguably, the high specificity in the context of differentiating bvFTD from PPD can be of more relevance than a high sensitivity. However, although the performance of NfL surpasses that of GFAP in distinguishing between bvFTD and late-onset PPD, its sensitivity requires enhancement to meet the standard for an ideal biomarker consistently exhibiting sensitivity and specificity values exceeding 80% for effective clinical and trial application.^31^ To date, only one study has achieved such thresholds in biomarker performance when differentiating bvFTD from PPD, in which the combination of GFAP and NfL yielded a sensitivity of 83.3% and specificity of 87.9%.^22^ This lack of sensitivity of NfL (and GFAP) in differentiating between sporadic bvFTD and late-onset PPD shows the necessity of finding a disease-specific biomarker for bvFTD.

Within a subset of sporadic bvFTD cases, our study revealed a significant correlation between elevated serum NfL levels at baseline and a shortened disease duration after blood sampling (ie, time between baseline visit and time of death), implicating a prognostic value of NfL in the context of sporadic bvFTD. This finding should be interpreted with caution because we cannot rule out the presence of reporting bias. In other words, it is possible that we only have mortality data available for a subset of bvFTD patients who declined more rapidly. Moreover, we could not find a significant correlation between GFAP level and disease duration, which contradicts previous studies that found correlations between serum GFAP levels and longitudinal brain atrophy rates, survival time and executive functioning.^22 32^ It should be noted that while disease duration, brain atrophy, mortality rate and neuropsychological scores are often considered as indicators of disease severity, they actually measure distinct aspects of disease progression in FTD. Moving forward, it is important to explore correlations between biomarker levels and various aspects of disease severity that can inform disease progression models.

Interestingly, we observed significant sex differences in sporadic bvFTD. NfL and GFAP levels were significantly higher in females than males, and sex had, in addition to age, a significant effect on NfL and GFAP levels. The observed sex difference aligns with a prior study which also noted elevated serum GFAP levels in females compared with males with bvFTD.^32^ In contrast, a meta-analysis showed that in healthy controls, multiple sclerosis, AD, vascular dementia and Parkinson’s disease, males had higher CSF NfL levels compared with females.^33^ Sex-linked differences specific to sporadic bvFTD have been reported.^34^ The observed sex difference might be explained by a higher cognitive reserve in females with FTD. Illàn-Gala *et al* (2021) found that females with bvFTD yield higher atrophy burden in brain regions affected in bvFTD compared with males despite similar disease severity and clinical presentation.^35^ Given that brain atrophy scores correlate with elevated NfL and GFAP levels,^22 36 37^ it is conceivable that higher atrophy burden in females results in higher levels of NfL and GFAP. The increases in NfL with ageing are well known, and interfaces to provide reference values of NfL levels in relation to age have been established.^38^ Nevertheless, the clinical implications of the observed sex difference remain uncertain, underscoring the relevance of considering sex in future biomarker analysis in sporadic bvFTD and the development of reference values interfaces.

Our major strength is that this is the largest multicentre study investigating the diagnostic value of NfL and GFAP in a well-characterised retrospective study cohort (DIPPA-FTD) including only sporadic bvFTD and late-onset PPD. This study, however, has its limitations. First, 12 cases with positive AD biomarkers were included in the analyses. In accordance with clinical criteria, a probable diagnosis of bvFTD should only be considered when there are no biomarkers indicative of AD.^1^ Sensitivity analyses, which excluded the 12 cases with AD positive biomarkers, yielded consistent results with our primary findings with one exception observed in the correlation analysis between disease duration postblood sampling and GFAP levels in the deceased bvFTD subgroup. Here, higher logGFAP values exhibited a significant correlation with shorter disease duration (n=51, r=−0.29, p=0.043) where primary analysis did not find a significant correlation (n=56, r=−0.26, p=0.053). Second, the retrospective design of this study withheld us from correcting for biological features, other than age and sex, known to affect biomarker levels such as body mass index and creatinine.^23 39^ Similarly, the potential influence of these biological factors on biomarker levels among the outliers within our cohort or site-specific differences (eg, different prevalence in heart or renal disease) remains unknown. Indeed, NfL and GFAP levels were significantly elevated in the Italian samples compared with other sites. A sensitivity analysis excluding these samples confirmed that our findings remained consistent (see online supplemental material 9 and 10). The prospective DIPPA-FTD cohort^25^ will give the opportunity to investigate the possible effect of these factors, including any potential biases introduced by the multicentre retrospective design, on both NfL and GFAP. Finally, pathology was only available for a small subset of 11 bvFTD cases; therefore, we cannot be completely certain of a sporadic bvFTD and PPD diagnosis. Nonetheless, it is important to note that patients were included in the DIPPA-FTD database only if expert clinicians were certain about the diagnosis, many of whom had follow-up assessments, and if genetic testing was performed and shown to be negative, reducing the chance of misdiagnoses. Additionally, individuals classified as having possible bvFTD, who are known to have the most diagnostic instability,^5^ were excluded.

Future directions should include sampling NfL and GFAP longitudinally in combination with different measurements of prognosis to enable the possibility to investigate the prognostic value of NfL and GFAP in more depth and to inform disease progression models. Furthermore, while the impact of age on NfL and GFAP is well established, the sex differences observed in sporadic bvFTD in this study highlight the importance of considering sex as a factor in future biomarker analyses. Moreover, it would be of interest to replicate earlier findings, where NfL and GFAP were shown to differentiate between FTLD-TAU and FTLD-TDP, once more pathological data become available.^40^ While GFAP was not a significant contributor, the potential utility of codetermination of GFAP next to NfL in understanding pathophysiological mechanisms and distinguishing underlying pathology should not be completely dismissed.

## Supplementary material

10.1136/bmjno-2024-001007 online supplemental file 1

## Data Availability

Data are available upon reasonable request.
