# Supplementary material for: Serum neurofilament light is superior to glial fibrillary acidic protein to distinguish sporadic frontotemporal dementia from late-onset primary psychiatric disorders: a retrospective DIPPA-FTD study
Source: BMJ Neurol Open. 2025 Jun 1;7(1):e001007. doi: 10.1136/bmjno-2024-001007 (PMC12142094; doi:10.1136/bmjno-2024-001007)
Supplement: online supplemental file 1 [file bmjno-7-1-s001.docx]

**Supplementary Material 1**

**Figure S1A. Passing Bablok regression NfL**


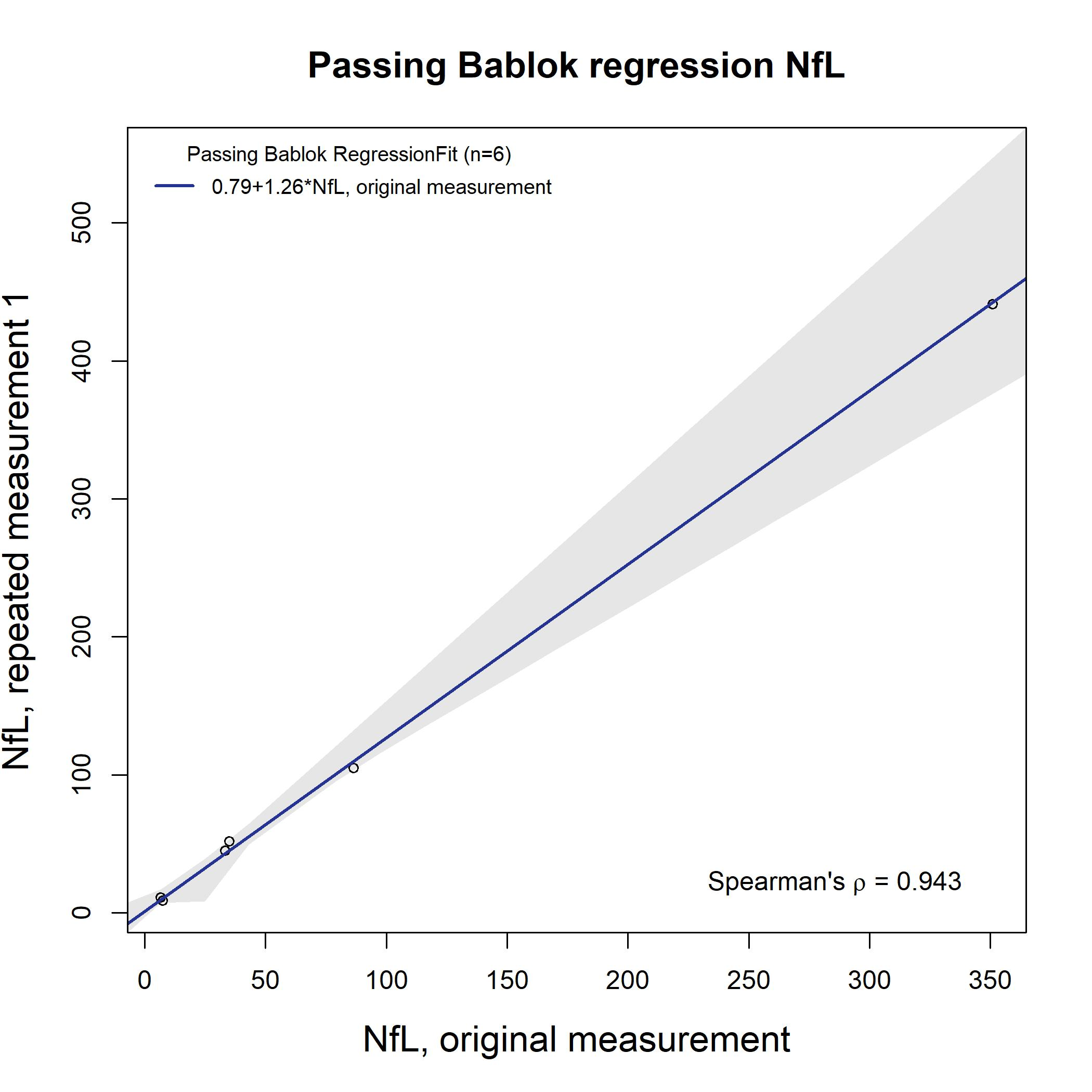


**Figure S1B. Passing Bablok regression GFAP**


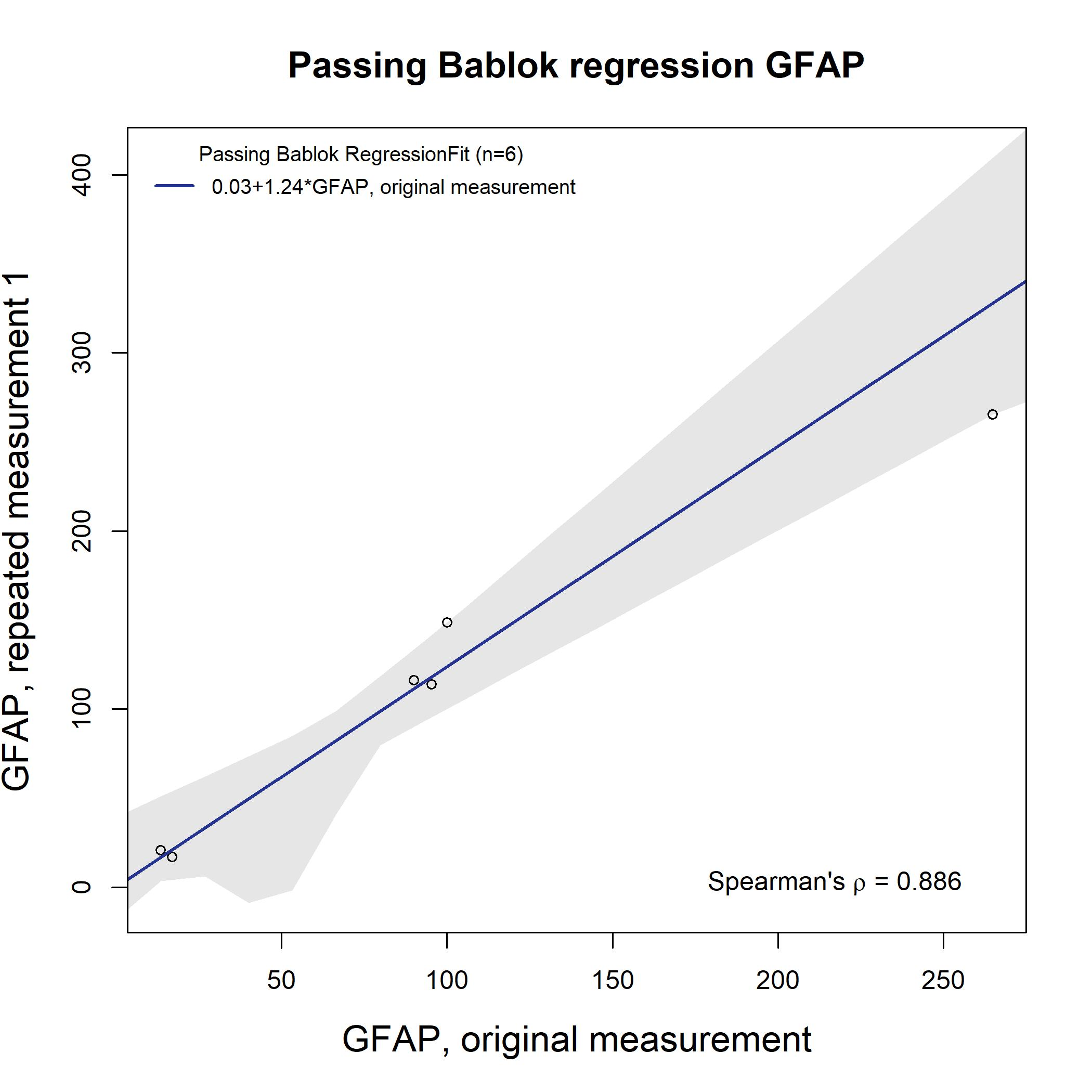


**Supplementary Material 2**

**S2.1 NfL and GFAP values**

|  | **Sporadic bvFTD** | **PPD** | **p-value** |
| --- | --- | --- | --- |
| N, % total | 275 (77.0) | 82 (23.0) | n.a. |
| NfL pg/mL, median (IQR) | 33.3 (19.6-49.6) | 12.2 (9.8-17.9) | <0.001^a^ |
| GFAP pg/mL, median (IQR) | 124.5 (83.5-181.6) | 68.9 (50.6-95.0) | <0.001^a^ |
| GFAP/NfL ratio, median, (IQR) | 4.0 (2.6-6.0) | 5.4 (3.7-7.3) | <0.001^a^ |
| NfL pg/mL, mean (SD) | 38.5 (24.5) | 14.4 (6.3) | n.a. |
| Log(NfL) pg/mL, mean (SD) | 3.5 (0.6) | 2.6 (0.4) | <0.001^b^ |
| GFAP pg/ml, mean (SD) | 144.0 (80.2) | 76.2 (35.1) | n.a. |
| Log(GFAP) pg/ml, mean (SD) | 4.8 (0.6) | 4.2 (0.5) | <0.001^b^ |

bvFTD: behavioural variant of frontotemporal dementia, GFAP: Glial fibrillary acidic protein, IQR: interquartile range, PPD: primary psychiatric disorder, NfL: Neurofilament light, NOS: not otherwise specified, SD: standard deviation, OCD=obsessive compulsive disorder.

^a^ Mann Whitney U test

^b^Independent student T-test

**S2.2 Boxplot of NfL values separate per clinical PPD subgroup**


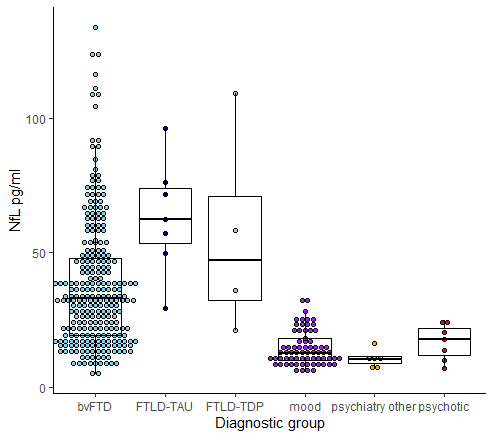


**S2.3 Boxplot of GFAP values separate for PPD subgroup**


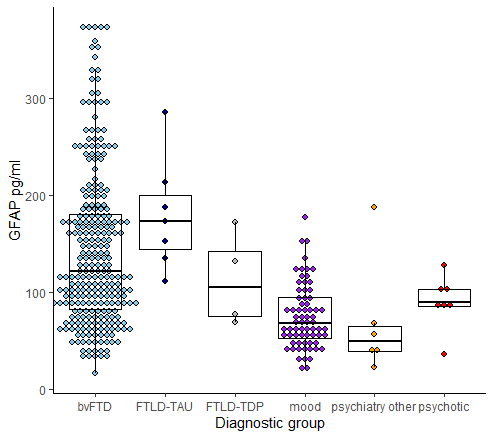


**Supplementary Material 3**

**S3. Scatterplot logNfL and age at sampling**


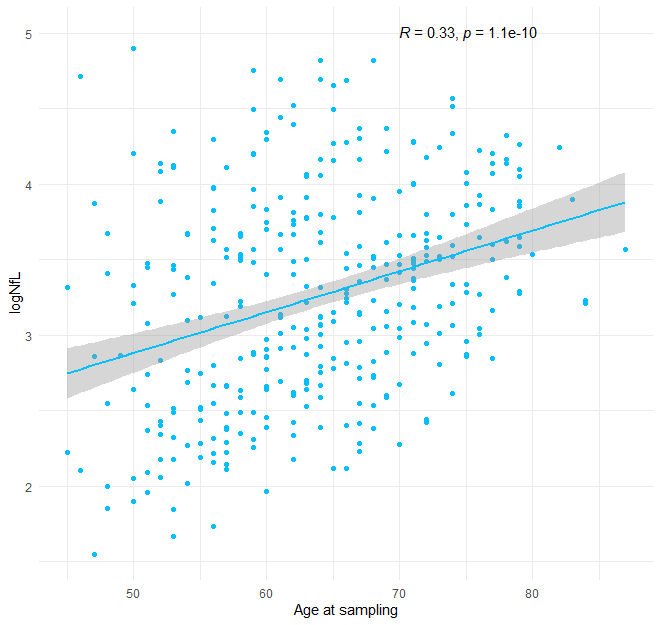


**Supplemantary Material 4**

**S4. Scatterplot logGFAP and age at sampling**


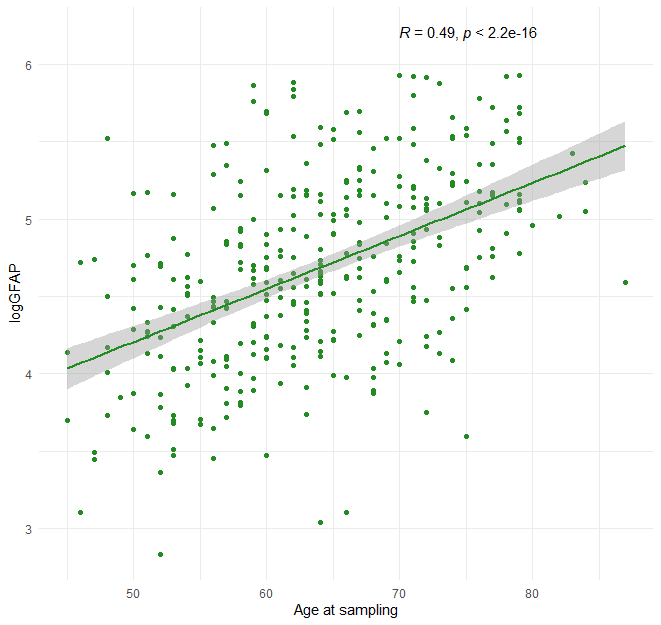


**Supplementary Material S5**

**S5. Scatterplot of logNfL and age at blood sampling, stratified by diagnostic group**

A. Sporadic bvFTD group


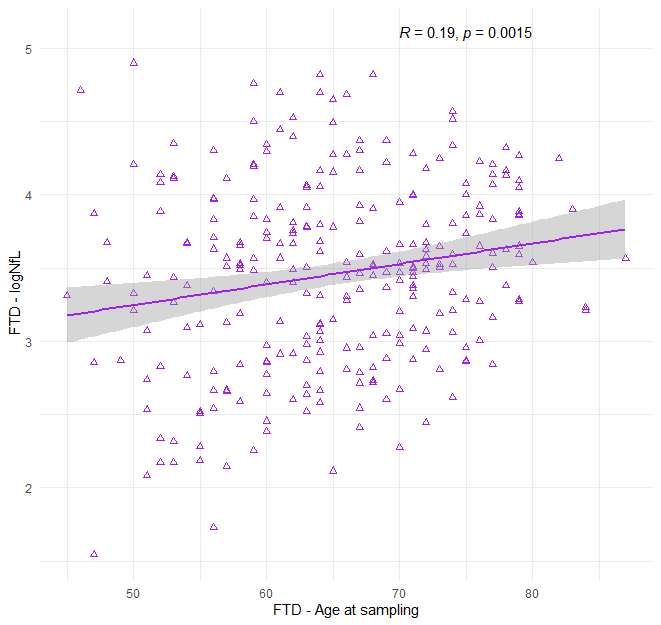


B. PPD group


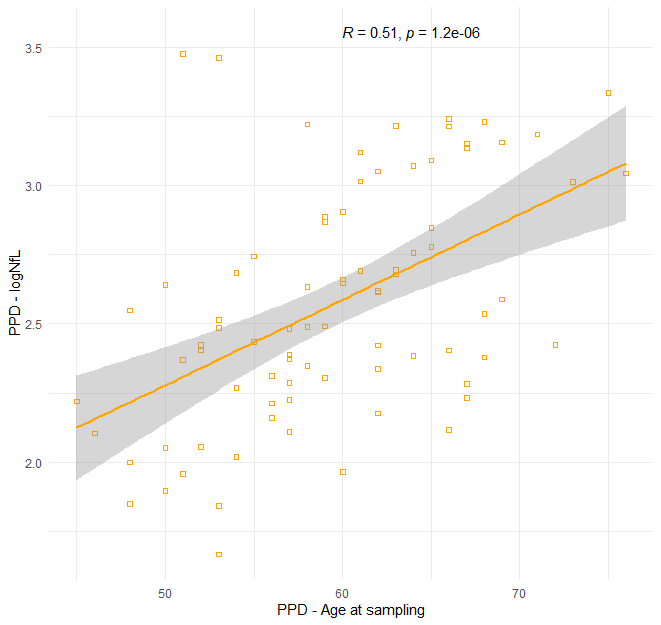


**Supplementary Material 6**

**S6. Scatterplot of logGFAP and age at blood sampling, stratified per diagnostic group**

A. Sporadic bvFTD group


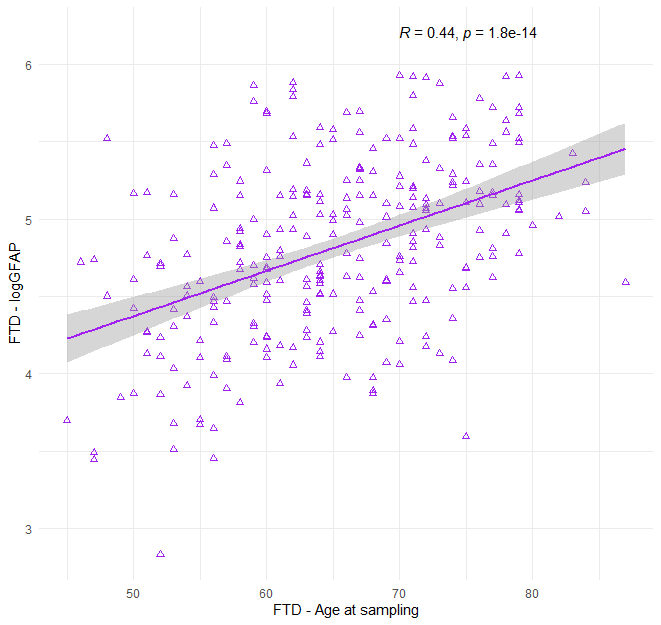


B. PPD group


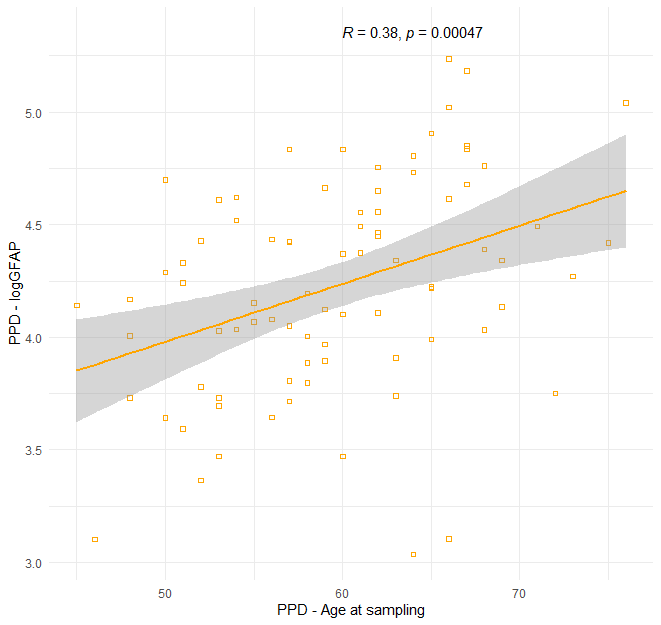


**Supplementary Material 7**

**Figure S7. Boxplots of serum NfL and GFAP stratified for diagnosis**

**
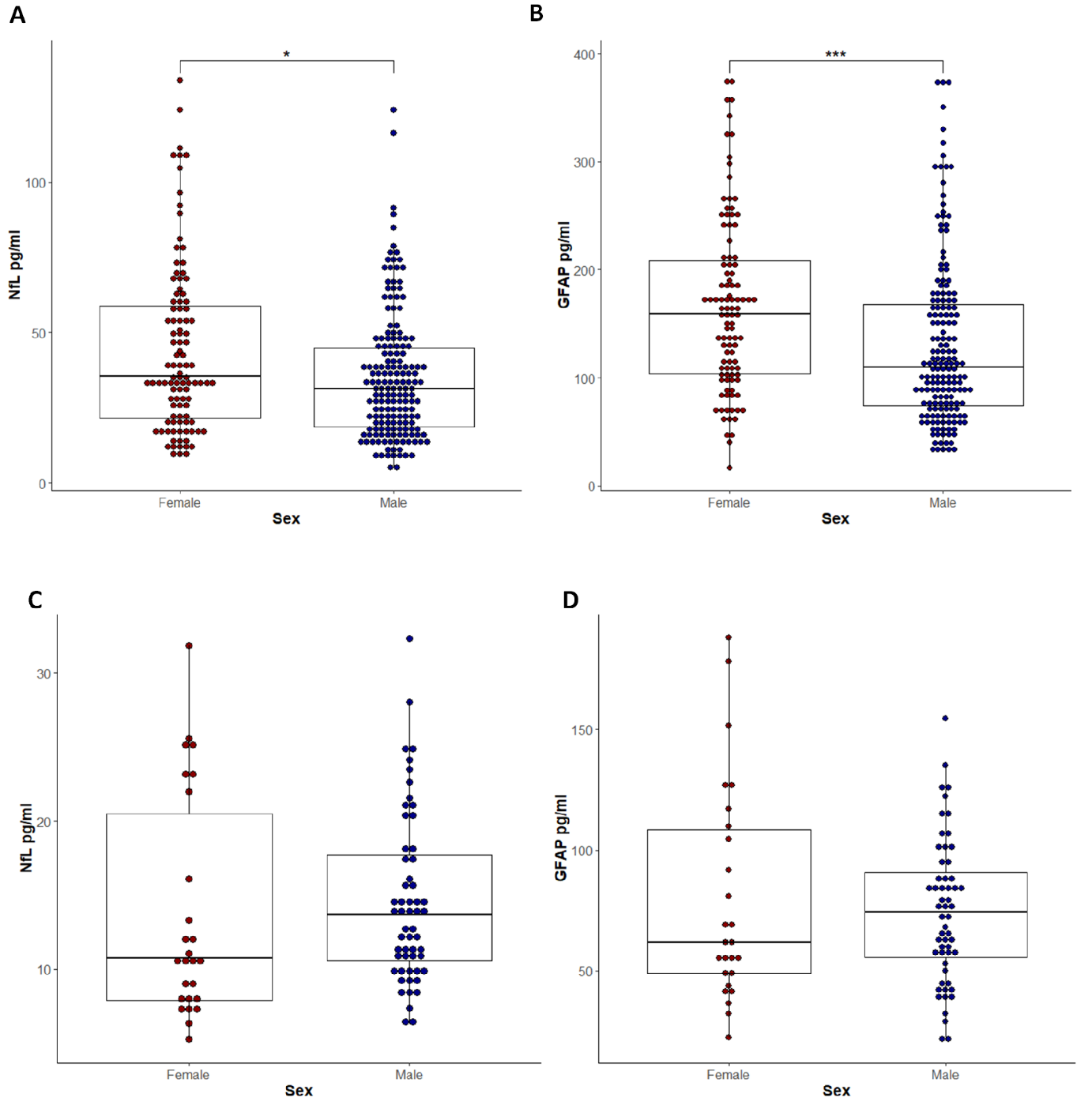
**

**(A)** serum NfL levels of females and males in the bvFTD group, females with bvFTD had a significant higher NfL level (median NfL 35.5, IQR 21.6-58.8) than males with bvFTD (median NfL 31.2, IQR 18.6-44.9, Mann-Whitney U, p-value = 0.015). **(B)** serum GFAP levels of females and males in bvFTD group, GFAP levels were significantly higher in females with bvFTD (median GFAP 159.4, IQR 103.8-208.3) compared to males with bvFTD (median GFAP 109.8, IQR 74.3-167.6, Mann Whitney U, p-value<0.001). **(C)** serum NfL levels of females and males in the PPD group, there was no significant differences in median NfL levels between females with PPD (median NfL 10.7, IQR 7.9-20.5) and males with PPD (13.7, IQR 10.6-17.8, Mann-Whitney, p-value = 0.156). **(D)** serum GFAP levels of females and males in the PPD group, there was no significant difference in median GFAP levels between PPD females (median GFAP 61.6, IQR 48.8-108.5) and PPD males (median GFAP 74.4, IQR 55.5-90.7, Mann-Whitney U, p-value = 0.800).

FTD: sporadic behavioural variant of frontotemporal dementia, GFAP: Glial fibrillary acidic protein, PPD: primary psychiatric disorder, NfL: Neurofilament light. *: statistically significant.

**Supplementary Material 8**

**S8. Output logistic regressions and ROC curves per model**

A. Model (i) NfL


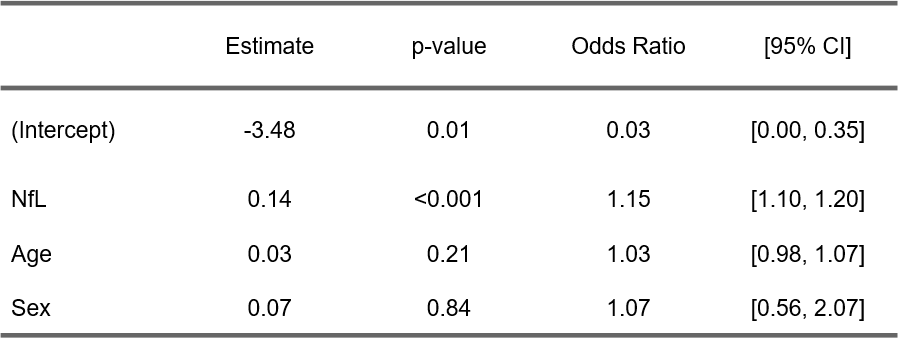


Diagnosis: PPD (0), FTD (1). Sex: male (0), female (1)

B. Model (ii) GFAP

|  | Estimate | p | Odds Ratio | [95% CI] |
| --- | --- | --- | --- | --- |
| (Intercept) | -2.45 | 0.03 | 0.09 | [0.01, 0.75] |
| GFAP | 0.02 | <0.001 | 1.02 | [1.01, 1.03] |
| Age | 0.03 | 0.17 | 1.03 | [0.99, 1.07] |
| Sex | -0.16 | 0.61 | 0.85 | [0.47, 1.57] |

Diagnosis: PPD (0), FTD (1). Sex: male (0), female (1)

C. Model (iii) NfL+GFAP

|  | Estimate | p | Odds Ratio | [95% CI] |
| --- | --- | --- | --- | --- |
| (Intercept) | -2.71 | 0.04 | 0.07 | [0.00, 0.84] |
| NfL | 0.12 | <0.001 | 1.13 | [1.09, 1.19] |
| GFAP | 0.01 | 0.03 | 1.01 | [1.00, 1.02] |
| Age | 0.01 | 0.79 | 1.01 | [0.96, 1.05] |
| Sex | -0.11 | 0.75 | 0.90 | [0.45, 1.78] |

Diagnosis: PPD (0), FTD (1). Sex: male (0), female (1)

D. Model (iv) GFAP/NfL

|  | Estimate | p | Odds Ratio | [95% CI] |
| --- | --- | --- | --- | --- |
| (Intercept) | -3.75 | <0.001 | 0.02 | [0.00, 0.18] |
| GFAP/NfL | -0.17 | <0.001 | 0.85 | [0.78, 0.92] |
| Age | 0.09 | <0.001 | 1.10 | [1.06, 1.14] |
| Sex | 0.49 | 0.09 | 1.63 | [0.93, 2.94] |

Diagnosis: PPD (0), FTD (1). Sex: male (0), female (1)

**Supplementary Material 9**

**S9. Site specific differences**

**Table 9A.** Distribution diagnosis per site.

|  | Sporadic bvFTD, n | PPD, n |
| --- | --- | --- |
| Amsterdam Dementia Cohort | 113 | 64 |
| FRONTIER Brain & Mind Centre | 62 | 10 |
| Technical University of Munich | 54 | 1 |
| University of Milan | 45 | 7 |

**Table 9B. Demographic and biomarker information per DIPPA site.**

|  | Amsterdam Dementia Cohort | FRONTIER Brain & Mind Centre | Technical University of Munich | University of Milan | p-value |
| --- | --- | --- | --- | --- | --- |
| Sporadic bvFTD, n | 113 | 62 | 54 | 45 | n.a. |
| PPD, n | 64 | 10 | 1 | 7 | n.a. |
| Age, mean (SD) | 62.3 (7.4) | 61.7 (8.3) | 63.8 (9.0) | 71.7 (8.1) | 2.113e-10 |
| FTD - NfL median (IQR) | 33.6 (29.1) | 25.3 (33.3) | 30.6 (23.7) | 40.6 (23.7) | 0.003 |
| PPD – NfL median (IQR) | 12.7 (10.2) | 10.1 (6.2) | n.a. | 10.8 (5.5) | 0.159 |
| FTD – GFAP median (IQR) | 114.0 (90.5) | 114.0 (101.0) | 103.0 (93.5) | 186.0 (99.1) | 3.907 e-08 |
| PPD – GFAP median (IQR) | 67.3 (45.0) | 65.4 (37.2) | n.a. | 80.7 (39.9) | 0.502 |

**Supplementary Material 10**

**S10. Sensitivity analysis. Samples from University of Milan excluded from analysis**

|  | **Sporadic bvFTD** | **PPD** | **p-value** |
| --- | --- | --- | --- |
| N, % total | 229 | 75 | n.a. |
| Male, n | 148 | 52 | 0.545^a^ |
| Age at sampling, mean (SD) | 64.3 (8.1) | 59.5 (7.0) | <0.001^b^ |
| NfL pg/mL, median (IQR) | 31.3 (29.8) | 12.4 (8.4) | <0.001^c^ |
| GFAP pg/mL, median (IQR) | 111.0 (94.9) | 67.9 (44.5) | <0.001^c^ |
| GFAP/NfL ratio, median, (IQR) | 3.9 (3.3) | 5.3 (3.4) | <0.001^c^ |

bvFTD: behavioural variant of frontotemporal dementia, GFAP: Glial fibrillary acidic protein, IQR: interquartile range, PPD: primary psychiatric disorder, NfL: Neurofilament light, NOS: not otherwise specified, SD: standard deviation, OCD=obsessive compulsive disorder.

^a^Pearson Chi-square

^b^Independent student T-test

^c^Mann Whitney U test

**Table 10A**. Model (i) NfL

|  | Estimate | p | Odds Ratio | [95% CI] |
| --- | --- | --- | --- | --- |
| (Intercept) | -2.93 | 0.03 | 0.05 | [0.00, 0.71] |
| NfL | 0.13 | 0.00 | 1.14 | [1.10, 1.20] |
| Age | 0.02 | 0.36 | 1.02 | [0.98, 1.07] |
| Sex | -0.02 | 0.96 | 0.98 | [0.50, 1.96] |

**Table 10B.** Model (ii) GFAP

|  | Estimate | p-value | Odds Ratio | [95% CI] |
| --- | --- | --- | --- | --- |
| (Intercept) | -1.94 | 0.10 | 0.14 | [0.01, 1.46] |
| GFAP | 0.02 | 0.00 | 1.02 | [1.01, 1.03] |
| Age | 0.02 | 0.34 | 1.02 | [0.98, 1.06] |
| Sex | -0.15 | 0.65 | 0.86 | [0.46, 1.64] |

**Table 10C.** Model (iii) NfL+GFAP

|  | Estimate | p | Odds Ratio | [95% CI] |
| --- | --- | --- | --- | --- |
| (Intercept) | 2.46 | 0.15 | 11.72 | [0.46, 356.9] |
| NfL | 2.90 | 0.00 | 18.11 | [6.70, 58.65] |
| GFAP | 0.57 | 0.05 | 1.77 | [1.02, 3.31 |
| Age | 0.00 | 0.93 | 1.00 | [0.95, 1.05 |
| Sex | -0.16 | 0.65 | 0.85 | [0.42, 1.73] |

**Table 10D.** Model (iv) GFAP/NfL

|  | Estimate | p | Odds Ratio | [95% CI] |
| --- | --- | --- | --- | --- |
| (Intercept) | -2.88 | 0.01 | 0.06 | [0.01, 0.53] |
| GFAP/NfL | -0.17 | 0.00 | 0.84 | [0.77, 0.92] |
| Age | 0.08 | 0.00 | 1.08 | [1.04, 1.12] |
| Sex | 0.44 | 0.15 | 1.55 | [0.86, 2.86] |

**
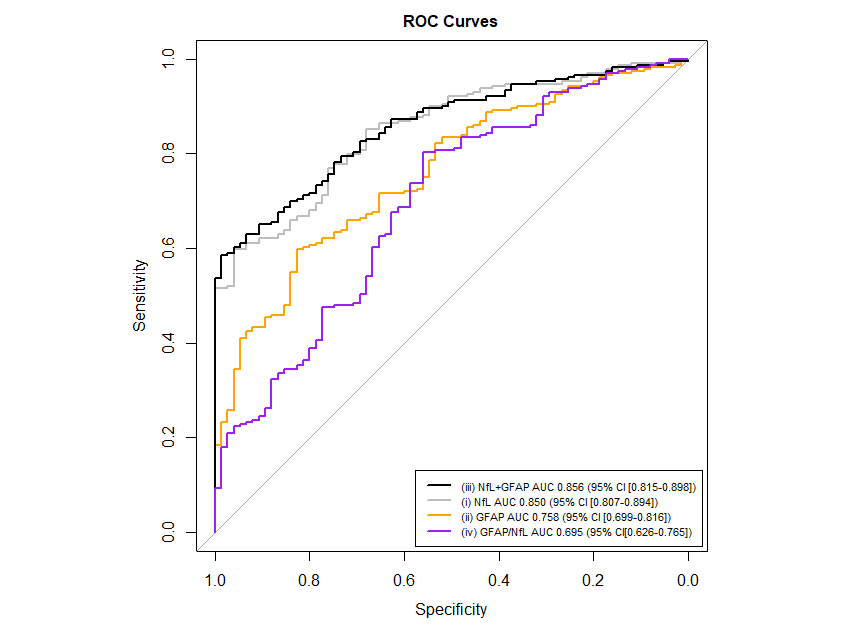
Figure 10A. ROC Curves for each model excluding samples from University of Milan**

Grey line: model (i) NfL. Orange line: model (ii) GFAP. Black line: model (iii) NfL+GFAP. Purple line: model (iv) GFAP/NfL. All models were adjusted for sex and age. The AUC value of (i) NfL was significantly higher than (ii) GFAP (p <0.001) and (iv) GFAP/NfL (p <0.001). The AUC of (i) NfL was lower but not significantly different from (iii) NfL+GFAP (p=0.314). AUC value of (ii) GFAP was significantly lower than (iii) NfL+GFAP (p <0.001) but not different from (iv) GFAP/NfL (p=0.155). The AUC of (iii) NfL+GFAP significantly differed from (iv) GFAP/NfL (p <0.001).

AUC: Area under the curve. CI: confidence interval. GFAP: Glial fibrillary acidic protein, NfL: Neurofilament light. ROC: receiver operating characteristic curve.
